# Regulation of lipid droplet dynamics in *Saccharomyces cerevisiae* depends on the Rab7-like Ypt7p, HOPS complex and V1-ATPase

**DOI:** 10.1242/bio.20148615

**Published:** 2015-05-06

**Authors:** Isabelle Bouchez, Marie Pouteaux, Michel Canonge, Mélanie Genet, Thierry Chardot, Alain Guillot, Marine Froissard

**Affiliations:** 1 Institut Jean-Pierre Bourgin IJPB, UMR 1318 INRA, Saclay Plant Sciences, route de St Cyr (RD 10), 78026, Versailles cedex, France; 2 Institut Jean-Pierre Bourgin IJPB, UMR 1318 AgroParisTech, route de St Cyr (RD 10), 78026, Versailles cedex, France; 3 MICALIS PAPPSO, UMR 1319 INRA, Domaine de Vilvert 78352, Jouy-en-Josas cedex, France; 4 MICALIS PAPPSO, UMR 1319 AgroParisTech, Domaine de Vilvert 78352, Jouy-en-Josas cedex, France

**Keywords:** Ypt7p, Rab GTPase, Lipid droplet, Vacuole, V-ATPase

## Abstract

It has now been clearly shown that lipid droplets (LDs) play a dynamic role in the cell. This was reinforced by LD proteomics which suggest that a significant number of trafficking proteins are associated with this organelle. Using microscopy, we showed that LDs partly co-localize with the vacuole in *S. cerevisiae*. Immunoblot experiments confirmed the association of the vacuolar Rab GTPase Rab7-like Ypt7p with LDs. We observed an increase in fatty acid content and LD number in *ypt7Δ* mutant and also changes in LD morphology and intra LD fusions, revealing a direct role for Ypt7p in LD dynamics. Using co-immunoprecipitation, we isolated potential Ypt7p partners including, Vma13p, the H subunit of the V1 part of the vacuolar (H+) ATPase (V-ATPase). Deletion of the *VMA13* gene, as well as deletion of three other subunits of the V1 part of the V-ATPase, also increased the cell fatty acid content and LD number. Mutants of the Homotypic fusion and vacuole protein sorting (HOPS) complex showed similar phenotypes. Here, we demonstrated that LD dynamics and membrane trafficking between the vacuole and LDs are regulated by the Rab7-like Ypt7p and are impaired when the HOPS complex and the V1 domain of the V-ATPase are defective.

## INTRODUCTION

In yeast, animals and other organisms, storage lipids are maintained in the cytoplasm in specialized organelles called lipid droplets (LDs) or lipid bodies (Brasaemle and Wolins, 2012; Chapman et al., 2012; Murphy, 2012; Walther and Farese, 2012). These structures consist mainly of a core of neutral lipids (triacylglycerols and/or steryl esters) enclosed in a monolayer of phospholipids, and contain a number of proteins which vary considerably with the species (Beller et al., 2010; Hodges and Wu, 2010). In the last decade, proteomic and genetic studies of this compartment have shown that LDs appear as a complex dynamic organelle with a role in metabolism control and cell signaling.

The structure of LDs and associated proteins (Blanchette-Mackie et al., 1995; Brasaemle, 2007; Frandsen et al., 2001), their biogenesis as well as their involvement in neutral lipid metabolism (Athenstaedt and Daum, 2006; Buhman et al., 2001) are abundantly documented in the literature. The current model for LD biogenesis hypothesizes that LDs are derived from endoplasmic reticulum (ER) (Farese and Walther, 2009; Radulovic et al., 2013; Thiam et al., 2013). Indeed, in addition to close contact visualized by transmission electron microscopy (TEM), most of the enzymes involved in neutral lipid synthesis are located in the ER in the absence of LDs. When LDs are present, many proteins show a dual localization between LDs and ER, like the ancient ubiquitous protein 1 (Stevanovic and Thiele, 2013). Furthermore, it has been demonstrated that LDs were functionally connected to the ER in *S. cerevisiae* (Jacquier et al., 2011).

Many studies have also reported interactions between LDs and other intracellular organelles, like early endosome (Liu et al., 2007), peroxisome (Binns et al., 2006; Schrader, 2001) and mitochondria (Wang et al., 2011). The yeast vacuole is not commonly described as an organelle directly involved in lipid metabolism. The role of lysosome, the mammalian vacuole, in lipid metabolism has been confined until recently to the breakdown of extracellular lipoproteins after endocytosis (Liu and Czaja, 2013). However, a relationship between LDs and vacuoles or lysosomes has been observed in many organisms. In *Arabidopsis thaliana*, the interaction of LDs with the vacuole leads to the mobilization of storage lipids (Poxleitner et al., 2006). Several authors have also described the critical role of lipophagy in the regulation of storage and metabolism of lipids in mammalian cells, through the uptake of LDs by autophagosomes and the degradation of their lipids by acidic hydrolases in the lysosome (Liu and Czaja, 2013; Singh et al., 2009; Zhang et al., 2009). It has been shown that LDs were engulfed and degraded by the vacuole via a process morphologically resembling microautophagy in *S. cerevisiae* (van Zutphen et al., 2014). In addition to this mechanism, the turn-over of LDs could be mediated by lipolysis (Thiam et al., 2013).

The main puzzling problem concerning the interactions between LDs and intracellular organelles is the fact that LDs have a phospholipid monolayer and all other organelles a phospholipid bilayer. Two main hypotheses are assumed, being both not excluded: some authors proposed that interactions could occur via membrane contact sites (MCSs) leading to exchange of lipids, small molecules and ions, without fusion (Helle et al., 2013; Zehmer et al., 2009). Thus, authors hypothesized the presence of a MCS between early endosome and LDs. Other authors proposed a model involving a hemi-fusion like mechanism (Binns et al., 2006; Murphy et al., 2009).

However, the exact mechanisms of LD dynamics remain to be solved. Subsequent functional studies have revealed a tight link between LD morphology, phospholipid metabolism (Fei et al., 2011) and ER-Golgi trafficking (COPII and Arf1-COPI) (Beller et al., 2008; Gaspar et al., 2008; Hommel et al., 2010). These results highlight the role of trafficking pathways in LD dynamics. This was reinforced by LD proteome data which demonstrated that a non-negligible number of trafficking proteins are associated with this organelle (Athenstaedt et al., 2006; Grillitsch et al., 2011; Larsson et al., 2012; Liu et al., 2004; Yang et al., 2012; Zehmer et al., 2009). Nearly all the studies found various forms of Rab GTPase. They proposed that each Rab species on LDs could regulate interaction of LDs with a specific organelle. Thus, Rab 5 has been identified like the Rab regulating the interaction of the early-endosome with LDs (Liu et al., 2007) and Rab 18 the interaction between LDs and ER (Ozeki et al., 2005). Recently, a novel LD associated Rab has been identified, Rab40c, which could modulate the biogenesis of LDs from the ER or ER-derived membrane compartments (Tan et al., 2013). Lately, Rab 7 has been described as a key regulatory component of the lipophagy in hepatocytes through its recruitment to LDs (Schroeder et al., 2015).

In this study, we carried out proteomic, biochemical and microscopic approaches in order to identify new proteins associated with both vacuole and LDs, and involved in the dynamics of LDs. We focused on one Rab, the yeast Rab7-like Ypt7p, a marker of the vacuole and late endosome (Balderhaar et al., 2010) and on some V-ATPase subunits shown in our study as associated to LDs.

## RESULTS

### LDs and their relationship with the vacuole/late endosome via Ypt7p

**Fig. 1.**
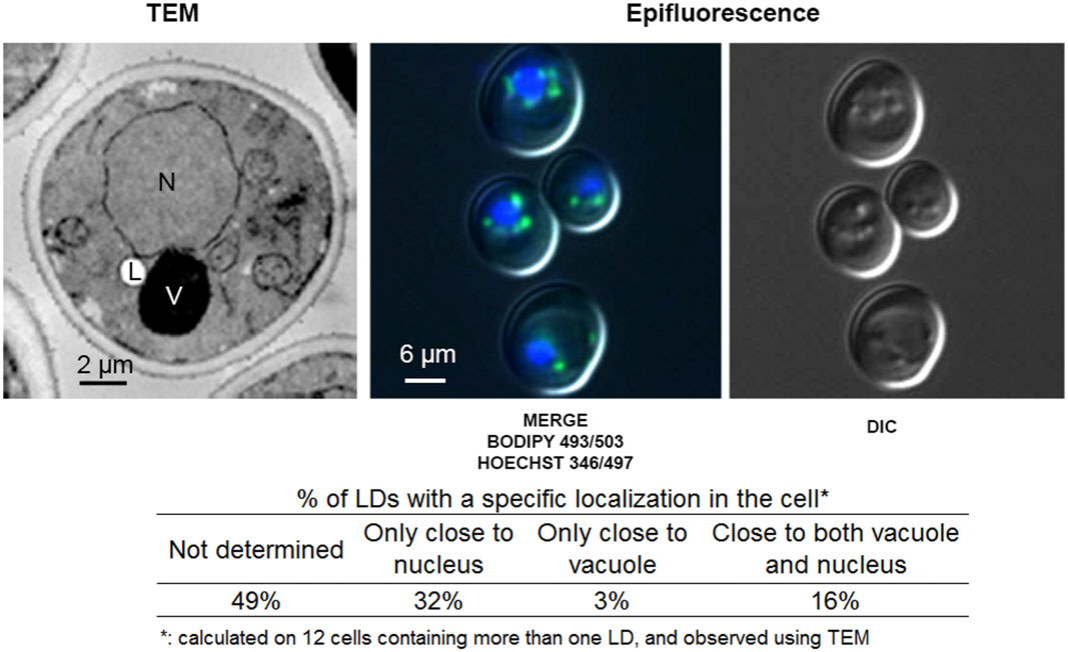
**LDs from *S. cerevisiae* are close to nucleus and vacuole.** Cells from WT were grown in YNB low C/N and observed using TEM or epifluorescence at the early stationary phase. LDs, stained with Bodipy 493/503, appear as small green spots and nuclei are stained blue with Hoechst 346/497. LDs can also be seen as small spheres in relief in DIC images. Percentage of LDs with a specific localization in the cell was calculated on 12 cells containing more than one LD, observed using TEM. L, LD; V, vacuole; N, nucleus. Differential interference contrast, DIC.

TEM and epifluorescence observations of *S. cerevisiae* wild-type (WT) cells (Fig. 1) showed that LDs are close to both the nucleus and vacuole (51% were in close proximity with nucleus alone, vacuole alone or both). Their proximity to the nucleus was expected as LDs are hypothesized to bud from the ER and were recently shown to be functionally connected to the ER membrane (Jacquier et al., 2011; Toulmay and Prinz, 2011). The proximity to the vacuole has not been investigated in yeast, out of nutrient restriction. Many organelles have been described in close contact with LDs, including the ER, endosomes, mitochondria and peroxisomes (for reviews, see Murphy et al., 2009; Zehmer et al., 2009) but these were functionally linked with fatty-acid (FA) metabolism. However, Rab7, a marker of the vacuole and late endosome, was consistently identified in proteomics of LDs (Murphy et al., 2009; Zehmer et al., 2009) and specifically on yeast LDs (Rab7-like Ypt7p) (Athenstaedt and Daum, 2006; Grillitsch et al., 2011). Thus, the question of whether there is a relationship between the vacuole/late endosome and LDs, via Ypt7p, was raised. Indeed, in yeast, this protein belongs to a highly conserved 11-member family of Ypt/Rab GTPase which is involved in the regulation of membrane recognition/docking processes (Hutagalung and Novick, 2011; Lachmann et al., 2011; Lazar et al., 2002). In particular, Ypt7p recruits the vacuolar homotypic fusion and vacuole protein sorting (HOPS) tethering complex which is required for fusion of autophagosomes, late endosomes and Golgi-derived protein 3 (AP-3) vesicles with the vacuole (Bröcker et al., 2010). In addition, Ypt7p could be involved in MCSs between LDs and the vacuole. In human, Rab7p interacts with Orp1lp, which regulates the MCSs between ER and late endosome (Johansson et al., 2005; Rocha et al., 2009).

**Fig. 2.**
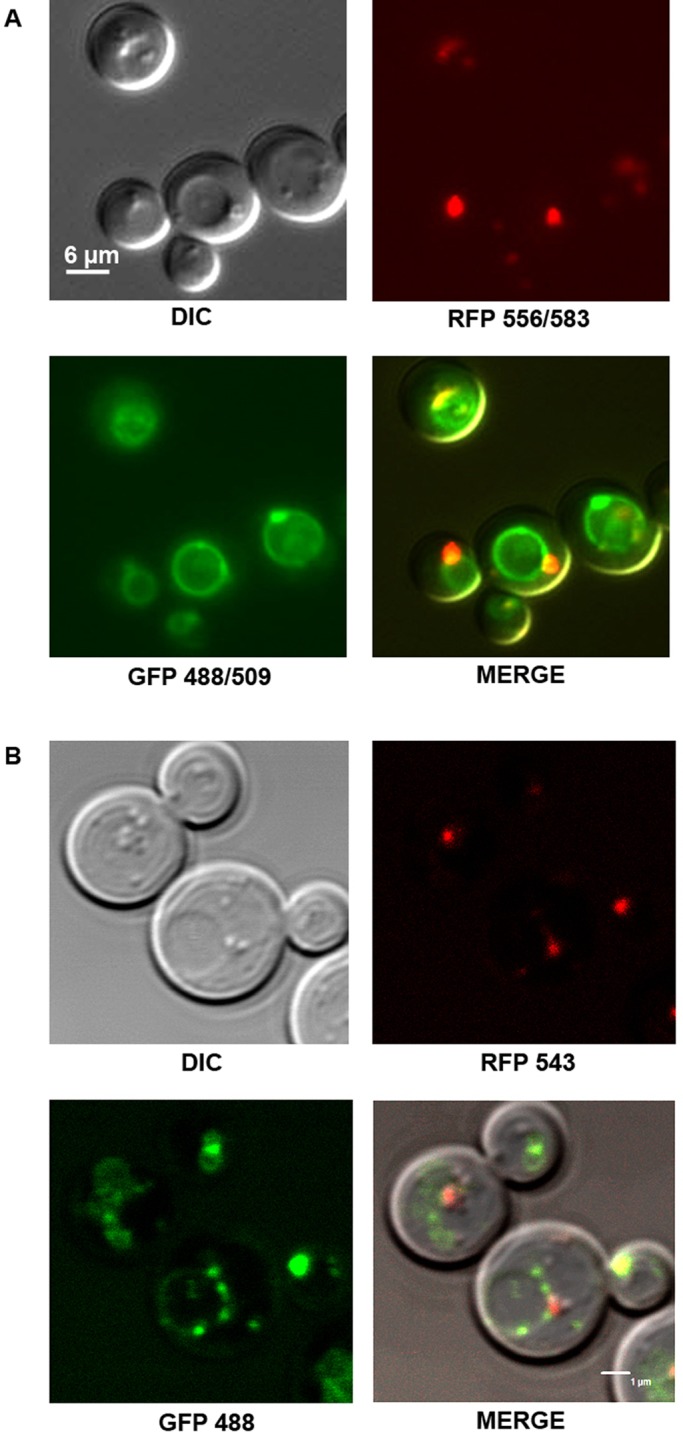
**Erg6p-RFP and GFP-Ypt7p in the *GFP-YPT7/ERG6-RFP* strain partly co-localize.** Cells were grown in YNB low C/N and observed at the early stationary phase. (A) Epifluorescence microscopy. (B) Confocal fluorescence microscopy. GFP-Ypt7p highlights the vacuolar membranes (green rings). Erg6p-RFP is targeted to LDs which appear as small red spots. LDs can also be seen as small spheres in relief mainly close to the vacuole in DIC images. Differential interference contrast, DIC.

To confirm preliminary microscopic observations, we modified a strain expressing Ypt7p tagged with the green fluorescent protein (GFP), a GFP-MYC-YPT7 strain (LaGrassa and Ungermann, 2005) by adding a DsRed fluorescent protein (RFP) tag to Erg6p, a sterol-Δ^24^-methyltransferase associated with LDs (Leber et al., 1994). Observations of GFP-YPT7/ERG6-RFP cells using epifluorescence microscopy (Fig. 2A) or confocal microscopy (Fig. 2B) showed a partial co-localization between GFP-Ypt7p and Erg6p-RFP. Overall, a clear relationship between the vacuole/late endosome and LDs was observed. This could involve Ypt7p as a trafficking protein between the two compartments.

To confirm our microscopic observations, we performed biochemical analysis and proteomics on LDs isolated by flotation on a discontinuous sucrose gradient.

### LDs and trafficking proteins

**Fig. 3.**
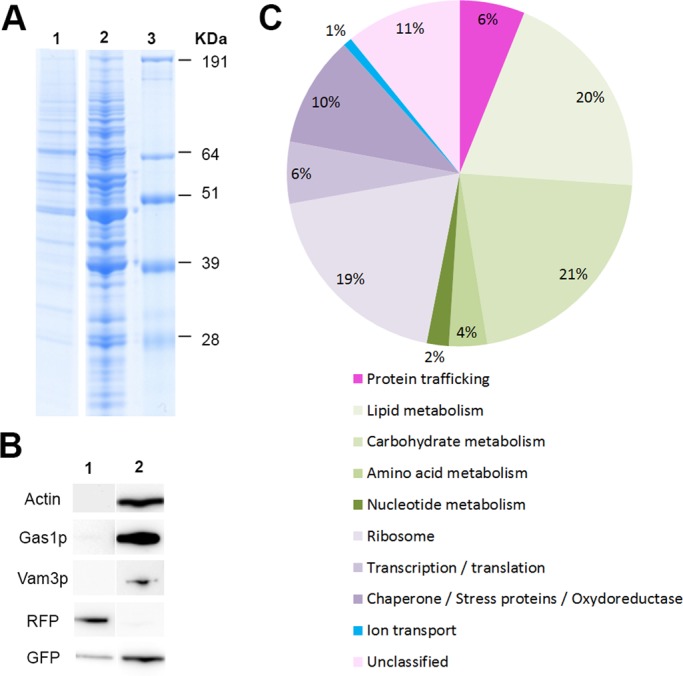
**Purified LDs contain numerous associated proteins, mainly belonging to metabolism, but also to trafficking pathways.** (A) SDS-PAGE analysis of purified LD protein extracts (1), total protein extract (2), molecular weight marker (3) after Coomassie staining. (B) SDS-PAGE then immunoblot analysis of purified LD protein extracts (1) and total protein extracts (2) with antibodies specific to the cytosol (anti-actin), plasma membrane (anti-Gas1p), vacuolar membrane (anti-Vam3p), Erg6p-RFP (anti-RFP) and GFP-Ypt7p (anti-GFP). (C) The 151 LD-associated proteins were classified into 10 groups according to the Saccharomyces Genome Database SGB (http://www.yeastgenome.org/). LDs were purified and total proteins extracted from the *GFP-YPT7/ERG6-RFP* strain.

In this study, we chose a mild procedure with a one-step sucrose gradient to purify the yeast LDs in order to avoid losing weakly-associated proteins (Vindigni et al., 2013). As a drawback, we increased the risk of identifying false associated proteins. For these reasons, we made functional validations via deletion strains for each protein we focused on before confirming their involvement in LD dynamics. The protein content of the purified LDs we prepared was analyzed using SDS-PAGE and compared to a total protein extract (Fig. 3A, lanes 1 and 2 respectively). To verify that we obtained a significant enrichment of LDs, the protein content of the fraction containing purified LDs was analyzed by immunoblotting using antibodies against markers of the cytosol (actin), plasma membrane (Gas1p) and vacuolar membrane (Vam3p), and compared to a total protein extract (Fig. 3B, lanes 1 and 2 respectively). We did not observe any reactivity with the purified LDs showing that there was no or limited contamination of the purified fraction by organelles other than LDs. In contrast, anti-RFP antibodies, which indicate the presence of Erg6p-RFP, strongly reacted with the purified LDs and not with the total protein extract. This observation confirms the enrichment of LDs in the floating fraction. Anti-GFP antibodies reacted against both purified LD proteins and the total protein extract, confirming the presence of GFP-Ypt7p on LDs.

Mass-spectrometry analysis of purified LDs from the GFP-YPT7/ERG6-RFP strain identified 151 associated proteins (Table 2 for a sub-set of the proteins identified based on their involvement in LD dynamics (lipid metabolism, trafficking proteins) and ion transport, and supplementary material Table S1 for the complete list) classified into ten groups based on function using the Saccharomyces Genome Database SGB (http://www.yeastgenome.org/) (Fig. 3C). We identified many proteins (19%) involved in lipid anabolism as previously described (Goodman, 2009). This suggests that LDs function as a cellular site for lipid synthesis or are involved in lipid mobilization, in addition to lipid storage (Athenstaedt et al., 1999; Yang et al., 2012). We also found numerous proteins from carbohydrate metabolism and, to a lesser extent, amino-acid and nucleotide metabolism. These results were more surprising but are consistent with recent results from other organisms such as *C. elegans* (Zhang et al., 2012) and *Rhodococcus sp RHA1* (Ding et al., 2012). Thus important bioprocesses could take place at the LD surface. We also identified ribosomal proteins and some translation factors. Although these types of proteins are often considered as contaminants, they were identified in many LD proteomic studies (Beller et al., 2010; Cermelli et al., 2006; Zhang et al., 2012). This suggests that proteins and not only lipids could be synthesized at the surface of LDs. Four proteins involved in ion transport were also identified. Interestingly, three belong to the peripheral V1 domain of the vacuolar (H^+^)-ATPase (V-ATPase subunits A, B and C). V-ATPases are multisubunit proton pumps responsible for the acidification of intracellular compartments in eukaryotic cells. Organelle acidification is involved, among others, in protein sorting in the biosynthetic and endocytic pathway and in the proteolytic activation of zymogen precursors (Forgac, 1999; Kane, 2006). It has also been shown that V-ATPase could be involved in sterol homeostasis, as deletion mutants corresponding to some V-ATPase subunits were susceptible to sterol inhibitors and induced effect on neutral-lipid synthesis (Fei et al., 2008b).

Finally, we identified a group of trafficking proteins, including two Arfs, one of their coatomers and Rabs. Among this latter group, we identified Ypt7p, which confirmed our microscopic observations and the relationship between LDs and the vacuole/late endosome, carrying both Ypt7p. We also identified three other Rabs known to be involved in trafficking between different organelles: Rab8-like Sec4p, between Golgi and plasma membrane; Rab1-like Ypt1p between ER and Golgi; Rab11-like Ypt31p in intra-Golgi transport. All have been previously described in association with LDs. These findings are consistent across almost all studies of the LD proteome from different organisms, including human, insect and yeast (Yang et al., 2012; Zehmer et al., 2009). This suggests that each Rab could regulate the interaction of LDs with a specific membrane system, perhaps via a transient inter-compartmental contact site (Liu et al., 2012; Zehmer et al., 2009) or a hemi-fusion like vesicular mechanism (Murphy et al., 2009).

### Ypt7p and its involvement in LD dynamics

**Fig. 4.**
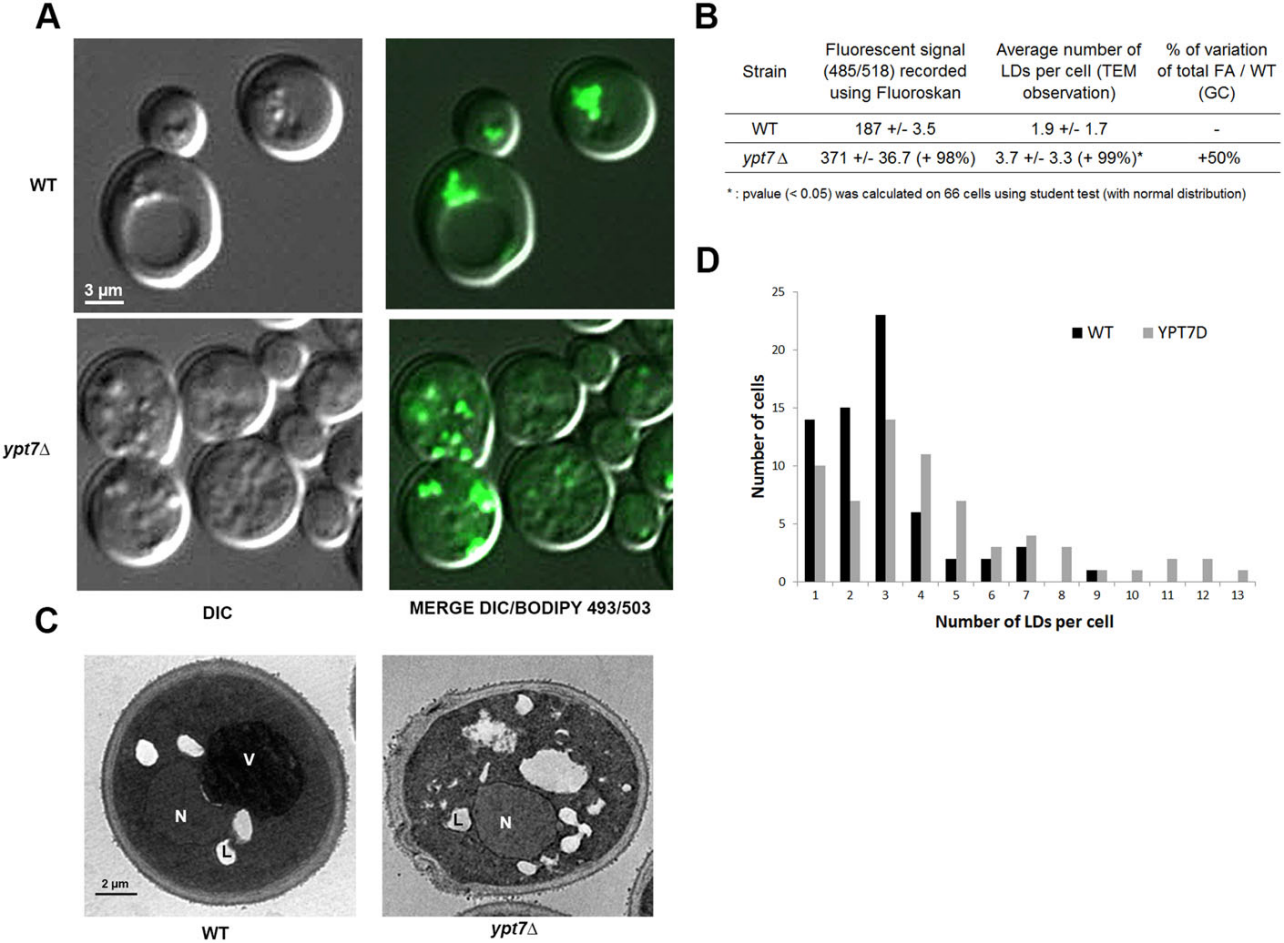
**Defect of Ypt7p function induces FA accumulation, LD proliferation and increase of fluorescence signal of Bodipy-stained cells when yeasts are grown in poor media.** Cells from *ypt7Δ* and WT strains were grown in YNB low C/N until the early stationary phase and LDs were stained with Bodipy 493/503. (A) Epifluorescence microscopy. LDs appear as small green dots in relief in the MERGE image. Differential interference contrast, DIC. (B) Strains (n=3) were analyzed using a Fluoroskan (485/518). The increase in *ypt7* mutant fluorescent signal compared to WT was calculated for an OD of 3.14. The average number of LDs per cell was determined on TEM images (n=66). The total FA content of *ypt7* strain (n=3) was determined using GC and compared to WT. (C) TEM. (D) The number of cells containing each from one to 13 LDs was calculated from TEM images (n=66 cells for each strain) to determine the LD number per cell repartition between WT and *ypt7p* strains. L, LD; V, vacuole; N, nucleus. *: *p* value (<0.05) was calculated on 66 cells using student test (with normal distribution).

To investigate the role of Ypt7p in LD dynamics, we performed genetic studies using one deletion strain (*ypt7Δ*). We confirmed the absence of Ypt7p on LDs from *ypt7* mutant by proteomic analyses (results not shown). The vacuoles appeared fragmented (Fig. 4A). This was expected as *ypt7* mutant is classified as class B *vps* with fragmented vacuoles (Haas et al., 1995). We evaluated the FA content of this strain, compared to WT, using gas chromatography (GC), under different conditions of nutrition [rich medium (YPG) and poor medium (YNB, low or high C/N)]. No change in total FA content was observed in YPG but we observed a 25% (high C/N) or 50% (low C/N) increase in FA in *ypt7* compared to WT (supplementary material Fig. S1).

FA analysis by GC gives an overview of the total lipid content of the cell. However, to confirm the specific involvement of LDs, we used neutral lipid staining of cells with Bodipy 493/503 combined with epifluorescence observations and fluorescence signal measurements. Such approaches using fluorescent neutral lipid specific dye for quantification are frequently used on LDs in microorganisms (Bae et al., 2013; Bozaquel-Morais et al., 2010; Radulovic et al., 2013; Sitepu et al., 2012).

Using epifluorescence microscopy (Fig. 4A) and TEM (Fig. 4C), the *ypt7* mutant strain was seen to contain a large number of LDs compared to the control strain. The average number of LDs per cell observed by TEM was increased of 99% whereas the repartition of the LD number per cell was clearly different (Fig. 4B,D). Ultrastructural observation of thin cell sections revealed contrasted LD morphologies between the two strains. These were altered and, in some cases, the fusion/internalization process between LDs or between LDs and the vacuole appeared “frozen”. The fluorescence signal of Bodipy-stained cells was also significantly higher for *ypt7* mutant compared to the control strain (+98% for *ypt7*Δ; Fig. 4B). Interestingly, similar observations were previously made in the *sec13-1* mutant (Gaspar et al., 2008). This strain, affected in COPII vesicle formation, has a very similar phenotype, with an increase in LD number and altered LDs associated with vacuoles. Together, these findings from the *ypt7* mutant show that this Rab GTPase is involved in LD dynamics. Thus, our next aim was to identify new actors associated both with vacuole and LDs and involved in the dynamics of these latter.

### A new actor involved in LD dynamics: Vma13p

To identify the LD proteins with which Ypt7p interacts, we carried out a co-immunoprecipitation (Co-IP) assay with the GFP-tagged protein. For this, a LD protein extract from the GFP-YPT7/ERG6-RFP strain was incubated with GFPm. Glycine- and LDS-eluted partners were analyzed using SDS-PAGE and silver nitrate staining or immunoblotted with anti-GFP antibodies (supplementary material Fig. S2). After comparison with controls (lanes 2 and 3), bands corresponding to potential Ypt7p partners (arrows) were cut out, digested and analyzed with LC-MS/MS.

Due to the high sensitivity of LC-MS/MS analysis, numerous proteins were identified in each band (all proteins are shown in supplementary material Table S2). First of all, we identified Gdi1p, which is the known GDP-dissociating factor for Ypt7p (Haas et al., 1995). This showed that the Co-IP experimental conditions were gentle enough to maintain interactions. However, some identified proteins could be non-specific partners or could be true Ypt7p partners, but not involved in LD dynamics. Thus, we selected one potential partner, Vma13p, the H subunit of the V-ATPase proton pump, for further characterization in order to highlight its effect on LDs and neutral lipid storage in yeast. It was chosen due to its vacuolar localization and with regard to our proteomic results.

**Fig. 5.**
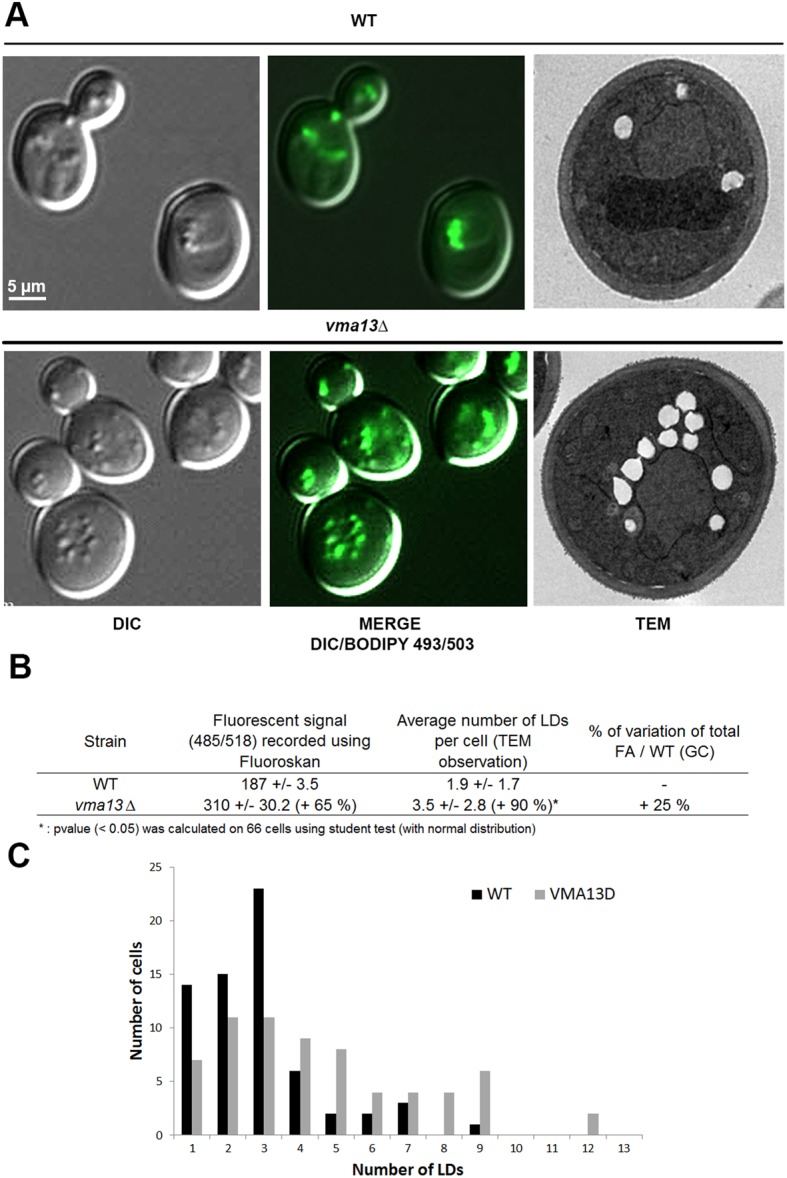
**Defect in Vma13p, one potential Ypt7p partner identified by co-immunoprecipitation, induces FA accumulation, LD proliferation and increase of fluorescence signal of Bodipy-stained cells.** Cells from *vma13*Δ and WT were grown in YNB low C/N until the early stationary phase and LDs were stained with Bodipy 493/503. (A) Epifluorescence microscopy and TEM. LDs appear as small green dots in relief in the MERGE image. Differential interference contrast, DIC. (B) Strains (n=3) were analyzed using Fluoroskan (485/518) after Bodipy 493/503 staining. The increase in *vma13* mutant fluorescent signal compared to WT was calculated for an OD of 3.14. The average LD number per cell (n=66 cells) was determined from TEM images and the increase in the *vma13* mutant compared to WT was calculated. The total FA content of *vma13* strain (n=3) was determined using GC and compared to WT. (C) The number of cells containing each from one to 13 LDs was calculated from TEM images (n=66 cells for each strain) to determine the LD number per cell repartition between WT and *vma13* strains. *: *p* value (<0.05) was calculated on 66 cells using student test (with normal distribution).

Deletion strain for *vma13* gene was analyzed using GC, fluorescent signal measurement, TEM and epifluorescence microscopy (Fig. 5). LD staining with Bodipy revealed a large increase in LD number in *vma13*Δ cells which was confirmed by TEM observation (+90% of LD number for *vma13*Δ compared to WT, Fig 5A,B). Similarly, we observed an increase in total FA for *vma13*Δ (+25% compared to the WT strain). This was also correlated with an increase in the fluorescence signal of Bodipy-stained mutant cells compared to the control strain (+65%). The repartition of the LD number per cell between the two strains was also greatly different (Fig. 5C). Together, these results showed Vma13p was involved in the dynamics of LDs. This result led us to focus on some other V-ATPase subunits, identified in our proteomic data as associated to LDs, in order to identify other actors associated with both vacuole and LDs and involved in these dynamics.

### Involvement of the V-ATPase in LD dynamics

**Fig. 6.**
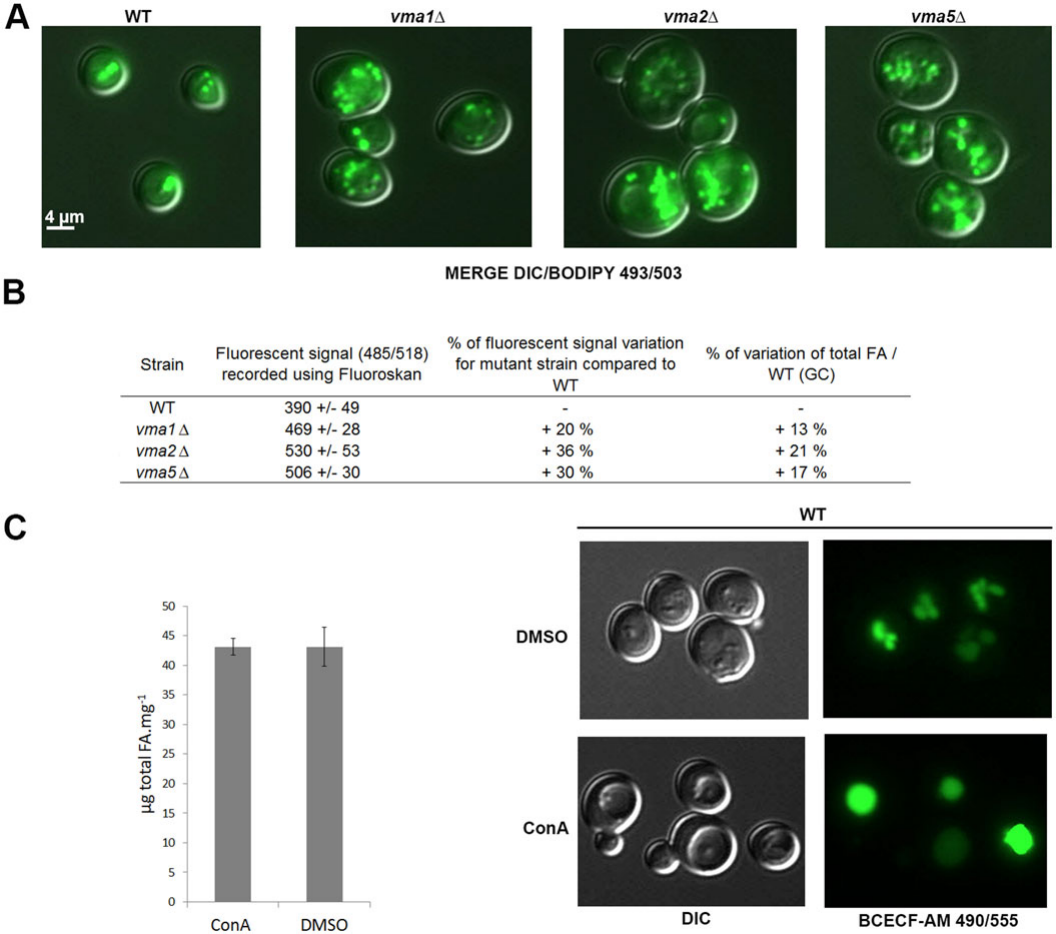
**Deletion of some of the VMA subunits of the peripheral part of the V-ATPase pump, but not chemical inhibition of the pump, has an effect on LDs.** Cells from WT, *vma1*Δ, *vma2*Δ and *vma5*Δ were grown in YNB low C/N until the early stationary phase and LDs were stained with Bodipy 493/503. (A) Epifluorescence microscopy. LDs appear as small green dots in relief in the MERGE image. Differential interference contrast, DIC. (B) Strains (n=3) were analyzed using a Fluoroskan (485/518) after Bodipy 493/503 staining. The increase in fluorescent signal from the mutant cells compared to WT was calculated for an OD of 15. The total FA content of mutant strains (n=3 for each) was determined using GC and compared to WT. (C) Cells from WT were grown in YNB low C/N until the exponential phase and incubated with 1 µM concanamycin A (ConA), an inhibitor of the V-ATPase, or DMSO, for 30 min, then collected for FA analysis by GC. Control of the experiment was done by epifluorescence analysis of the cells incubated with BCECF-AM 490/455, a vacuole marker, before ConA or DMSO treatment. Error bars: mean/s.d.

Deletion strains for the three V-ATPase subunits associated to LDs [subunit A (Vma1p), subunit B (Vma2p) and subunit C (Vma5p)] were analyzed using GC, fluorescence signal measurement and epifluorescence microscopy (Fig. 6). Compared to the WT strain, we observed an increase in total FA for *vma1*Δ (+13%), *vma2*Δ (+21%) and *vma5*Δ (+17%) (Fig. 6B). LD staining with Bodipy 493/503 revealed an increase in LD number, as for *ypt7* and *vma13* mutants (Fig. 6A). This was confirmed by the analysis of the fluorescence signal of Bodipy-stained mutant cells compared to the control strain (+20% for *vma1*Δ, +36% for *vma2*Δ and +30% for *vma5*Δ, Fig 6B). So, Vma1p, Vma2p and Vma5p were all involved in LD dynamics, as Vma13p was.

It is known that the deletion of any of the single copy V-ATPase subunit gene leads to a *vma*- phenotype, characterized by a complete loss of acidification of the vacuole and ATPase activity, inability to grow on neutral media and sensitivity to high calcium concentrations (Baars et al., 2007; Kane, 2006; Li and Kane, 2009). So, the increase in LDs in the *vma* mutants tested could be an indirect consequence of some of these defects, in particular the loss of acidification of the lumen. In order to answer this question, we used Concanamycin A, an inhibitor of the V-ATPase proton transport activity, to see if a chemical blocking of the pump activity has a similar effect on the dynamics of LDs than the deletion of some of its subunits. WT strains were grown in YNB until exponential phase with or without 1 µM of Concanamycin A for 30 min then collected and analyzed for FA by GC (Fig. 6C). Efficiency of Concanamycin A treatment was evaluated using fluorescent microscopy as previously described (Johnson et al., 2010). Cells were observed after staining with BCECF-AM and a characteristic phenotype due to reduced vacuole fission, i.e. large vacuole, was observed for cells treated with Concanamycin A (Baars et al., 2007). FA analysis using GC revealed no difference between treated and non-treated cells. These results showed that LD dynamics is not dependent on vacuolar V-ATPase function per se. Thus, the increasing of LDs in *vma* mutants could not be due to an indirect effect of the loss of vacuole function, but rather due to a direct effect of the loss of the subunits, for example by modifying relationships between these Vmap subunits and LDs.

### The HOPS pathway and LD dynamics

We investigated whether the relationship between LDs and the vacuole could occur via the vacuolar HOPS tethering complex, which is recruited by Ypt7p. This multisubunit tethering complex (MTC) contains six subunits: four shared with the CORVET tethering complex (Vps11p, Vps16p, Vps18p, Vps33p) and two specific subunits (Vps39p, Vps41p). Ypt7p acts as the Rab effector involved in the targeting, tethering and docking of vesicles trafficking to the vacuole (endosomes, AP-3 and autophagic vesicles) or between vacuoles (Bröcker et al., 2010). Interacting SNAREs (Vam3p, Vam7p, Vti1p, Nyv1p) localized on the vacuole are also involved during the two later steps, and for the fusion between the vesicle and the vacuole.

**Fig. 7.**
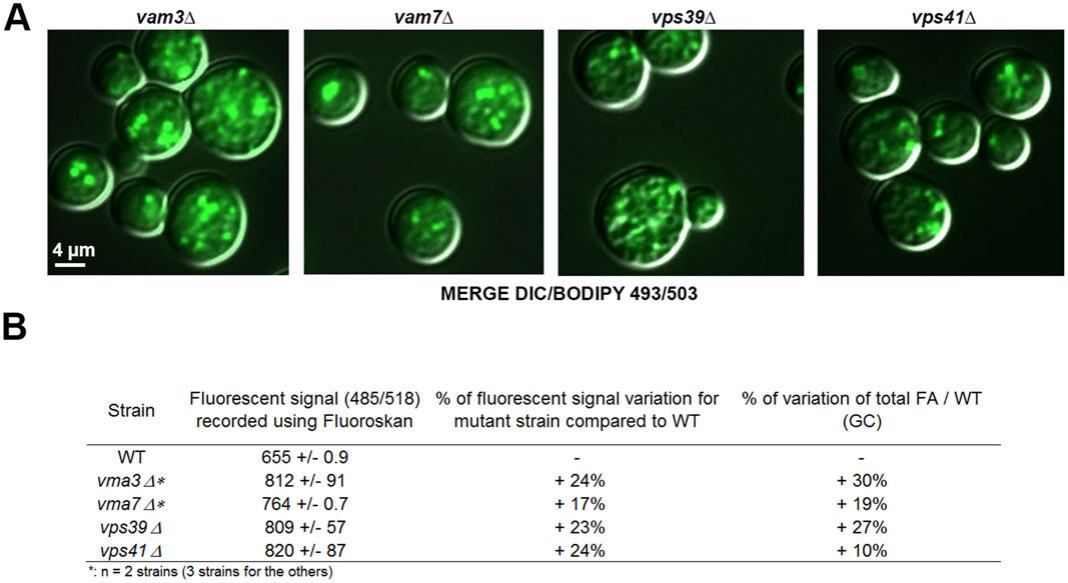
**Deletion of proteins from the HOPS pathway has a drastic effect on LDs.** Cells from WT, *vps39*Δ, *vps41*Δ, *vam3*Δ and *vam7*Δ were grown in YNB low C/N until the early stationary phase and LDs were stained with Bodipy 493/503. (A) Epifluorescence microscopy. LDs appear as small green dots in relief in the MERGE image. Differential interference contrast, DIC. (B) Strains (n=3 except * where n=2) were analyzed using a Fluoroskan (485/518) after Bodipy 493/503 staining. The increase in fluorescent signal from the mutant cells compared to WT was calculated for an OD of 20. The total FA content of mutant strains (n=3 for each) was determined using GC and compared to WT.

Due to the association of Ypt7p with LDs, we hypothesized that LDs could recruit Ypt7p and then the HOPS complex and associated SNAREs in order to interact with the vacuole. We followed the same strategy as previously described for the *vma* mutants and used deletion mutant strains for the proteins belonging specifically to the HOPS complex (*vps39*Δ and *vps41*Δ) and two vacuolar SNAREs involved in the tethering and docking of the vesicles carrying Ypt7p (*vam3*Δ and *vam7*Δ). As before, these strains were characterized using GC, epifluorescence microscopy and fluorescent signal measurement (Fig 7). The total FA content was significantly higher in the mutant cells compared to WT (Fig. 7B: +30% for *vam3*Δ, +19% for *vam7*Δ, +27% for *vps39*Δ and +10% for *vps41*Δ) and the vacuoles appeared fragmented, as seen for *ypt7*Δ. This phenotype was expected because the four proteins are required for the last step of vacuolar assembly and belong to the class II VAM genes, mutants of which are characterized by numerous small vacuoles (Nakamura et al., 1997; Wada et al., 1992). Bodipy 493/503 staining revealed a larger number of LDs in mutant strains compared to the WT and the fluorescent signal of these cells also significantly increased (+24% for *vam3*Δ, +17% for *vam7*Δ, +23% for *vps39*Δ and +24% for *vps41*Δ).

In conclusion, the absence of some proteins involved in the HOPS complex, as well as the absence of some subunits of the V1 part of the V-ATPas, led to an increase in FA and LDs in the cell. This confirms the relationship between LDs and the vacuole, and demonstrates that the vacuolar trafficking machinery and the HOPS complex are involved in LD dynamics.

## DISCUSSION

It is now well established that LDs are not only fat storage organelles but play a dynamic role in lipid homeostasis, probably managing lipid trafficking among membrane systems. In the present study, we showed, using TEM and epifluorescence microscopy that LDs are often in close contact with the vacuole in *S. cerevisiae*. Using colocalization, proteomic analysis and immunoblot experiments, we confirmed the localization of Ypt7p, a vacuole-associated protein, on LDs. This is consistent with previous studies as this protein is the most frequently identified Rab on LDs from different organisms (Athenstaedt and Daum, 2006; Ayciriex et al., 2012; Cermelli et al., 2006; Grillitsch et al., 2011; Zehmer et al., 2009; Zhang et al., 2012).

We observed that a defect in Ypt7p functions drastically increases the total FA content and the number of LDs in cells. It is well known that in *ypt7* mutants, vacuolar fusion processes are impaired and as a result trafficking pathways leading to the vacuole are also altered (Haas et al., 1995). LD accumulation could be the consequence of altered vacuole dynamics but our TEM observations revealed significantly altered LD morphology and intra LD contact and/or fusion, revealing a key role for Ypt7p in LD dynamics.

Hemi-fusion between LDs and vacuole has previously been described in plants during seed germination where the phospholipid monolayer of LDs, partly depleted of lipids, fuses partially with the vacuolar membrane (Huang, 1996; Wang and Huang, 1987). It was also proposed that LDs could make contact with bilayered organelles through a hemi-fusion mechanism in LD-peroxisome interaction (Binns et al., 2006). In this way, lipids and membrane-associated proteins that do not span the bilayer could exchange without mixing the neutral lipids with the lumen of the other organelle (Murphy et al., 2009). To investigate whether the LD-vacuole relationship involves the HOPS machinery, and thus membrane hemi-fusion like mechanisms, we studied the effect of the deletion of proteins from the HOPS complex on LD behavior. This complex is required for fusion events at the late endosome and the vacuole. HOPS complex mutants showed phenotypes similar to those of *ypt7* mutants. Defects in Vps39p, Vps41p or Vam7p, which are direct Ypt7p interactors in the HOPS complex (Brett et al., 2008; Ungermann et al., 2000; Wurmser et al., 2000) induced FA accumulation and an increase in LD number. Hemi-fusion mechanisms could, therefore, be involved in LDs-vacuole/late endosome contact, as hypothesized between LDs and peroxisome.

Hemi-fusion mechanisms would involve SNAREs. Here, we showed that two interacting SNAREs involved in the HOPS complex (Vma3p and Vma7p) had an effect on LDs dynamics. These SNAREs could act as the t-SNAREs, carried by vacuole membrane. The v-SNAREs associated to LDs remain to be identified. In the proteomic analysis of LDs presented in this article, we did not. However, SNAREs (Sso1p and Use1p) have already been identified in other proteomic analysis of *S. cerevisiae* LDs (Grillitsch et al., 2011). In mammals, it has been shown that the essential components of the SNARE-dependent fusion (α-NAP, SNAP23, syntaxin-5, VAMP4, SNAP25, and NSF) were present on ADRP-containing LDs and were involved in LD-LD fusion (Boström et al., 2007).

Other pathways, such as the macroautophagy and cytoplasm-to-vacuole (cvt) pathways, where organelles are enclosed in a double-membrane before fusing with the vacuole, bypass the question of bilayer/monolayer fusion, but take place upon nutrient restriction (Suzuki, 2013). Lipophagy was described as a regulator of lipid metabolism in mammalian cells (Singh et al., 2009; Zhang et al., 2009). In this process, autophagosomes containing LD are delivered to the vacuole/lysosome thought membrane fusion process. Recently, it was also published that LDs are submitted to another autophagy process resembling to microautophagy in yeast (van Zutphen et al., 2014). Furthermore the role of Rab7 in lipophagy was described in hepatocytes (Schroeder et al., 2015). The authors demonstrated the interaction of LDs with bilayer compartments, such as late endosomes and vacuole/lysosome through Rab7-positive membranous structures and “kiss-and-run”-like mechanism. These recent results obtained in mammals on the role of Rab7 in LD multiple fusion processes reinforce the data we obtained on the role of Ypt7 on LD dynamics in yeast.

Proteomic analysis showed that three subunits from the peripheral V1 domain of the V-ATPase were associated to LDs. Moreover, Vma13p, the H subunit of the peripheral V1 domain of the V-ATPase, was identified by Co-IP experiments as a potential Ypt7p partner on LDs. These results lead us to focus on the V-ATPase. Functional validation through biochemical and microscopic studies of a *vma13*Δ mutant demonstrated that the deletion of *VMA13* gene modifies the total FA content and the number of LDs. These results are in accordance with those published previously (Lin et al., 2009; Lin et al., 2012). Indeed, they showed that sterol level was increased in cell lacking Ste20p, protein which was identified as a partner of Vma13p. So, the lacking of Vma13p could likely also disturb the sterol homeostasis. This hypothesis was reinforced by further previously obtained results (Fei et al., 2008a). They led a large screen of *S. cerevisiae* mutants for abnormally in the number and morphology of LDs and identified *vma13* and some other *vma* mutants (*vma6*, *vma8* and *vma21*) as *mld* (*many* LDs) mutants. Moreover, in a genome wild analysis of sterol-lipid storage in *S. cerevisiae* (Fei et al., 2008b), it has been shown that several deletion mutants corresponding to some V-ATPase subunits were susceptible to sterol inhibitor. As not all the subunits seemed involved, they deduced that steryl ester storage was not dependent on V-ATPase function per se. The V1 sector contains three copies of the catalytic A subunit (Vma1p), which is responsible for ATP hydrolysis, three copies of B subunit (Vma2p) which is believed to play a regulatory role and six other subunits, whom C subunit (Vma5p) and H subunit (Vma13p) that appear to connect the catalytic head group with the membrane domain (V0 sector) (Kane, 2006). We showed that all of them, despite their distinct functions, were associated to LDs and involved in their dynamics. Conversely, the inhibition of the V-ATPase proton transport activity by Concanamycin A showed no effect on LD dynamics. Thus, these latter do not appear to be directly dependent on the V-ATPase function per se, but rather more related to a direct relationship between some *vma* subunits and LDs. This proton pump is responsible for the acidification of the vacuolar compartment and also plays a role in calcium and heavy metal homeostasis as well as in membrane traffic processes and sterol homeostasis (Fei et al., 2008b; Forgac, 1999; Kane, 2006). So, our results showed a link between LD dynamics and vacuole-linked processes through some subunits of the V1 sector of the V-ATPase.

Our results revealed that LDs interact with the vacuole and that Ypt7p, the HOPS complex and the V-ATPase are involved in these dynamics. It will now be interesting to clearly decipher the protein interaction network and the time course of events leading to the interaction between LDs and the vacuole.

## MATERIALS AND METHODS

### Yeast strains and growth conditions

The yeast strains used in this study are shown in Table 1. *GFP-MYC-YPT7* expressing the RFP-tagged Erg6p protein was constructed by inserting the REDSTAR2 coding sequence in frame at the 3′ of the ERG6 gene by homologous recombination as previously described (Janke et al., 2004) in the *GFP-MYC-YPT7* strain (LaGrassa and Ungermann, 2005). *ypt7*Δ strain was constructed by homologous recombination of specific DNA cassettes in the BY4741 strain as described previously (Janke et al., 2004). The BY4741 strain was transformed with the plasmid p416ADH-GFP (generous gift from R. Haguenauer-Tsapis, Institut Jacques Monod, Paris) to obtain the WT+p416GFP strain. Cells were grown either in poor medium containing 0.67% (w/v) yeast nitrogen base without amino acids and ammonium sulfate (YNB), supplemented with 5 g l^−1^ ammonium sulfate and 0.2% (w/v) casamino acids, with 2% (w/v) glucose as the carbon source (YNB low C/N), or in YNB supplemented with 0.5 g l^−1^ ammonium sulfate and 0.2% (w/v) casamino acids, with 4% (w/v) glucose as the carbon source (YNB High C/N), or in rich medium (YP) supplemented with 2% (w/v) glucose (YPG). All cultures were grown in conical flasks, containing 1/5 volume of medium, and incubated at 28°C in an orbital shaker with an agitation rate of 200 rpm.

**Table 1.**
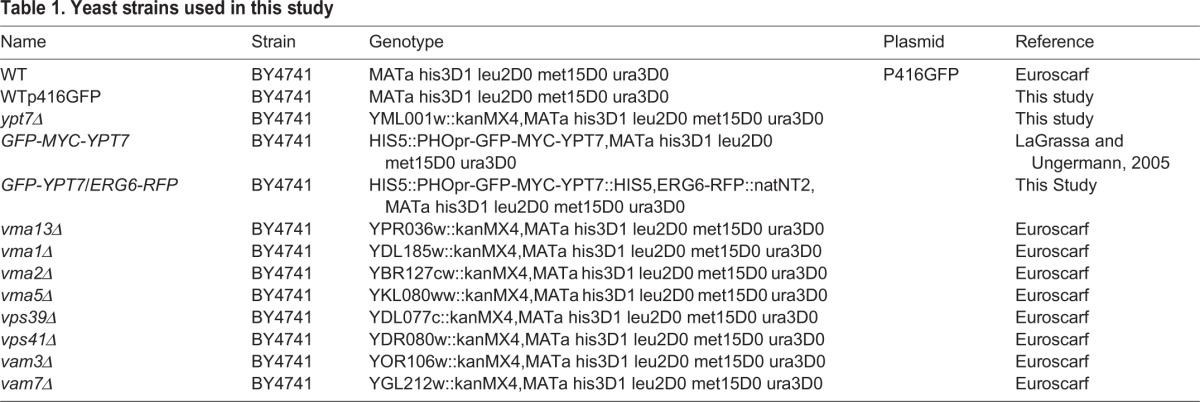
**Yeast strains used in this study**

**Table 2.**
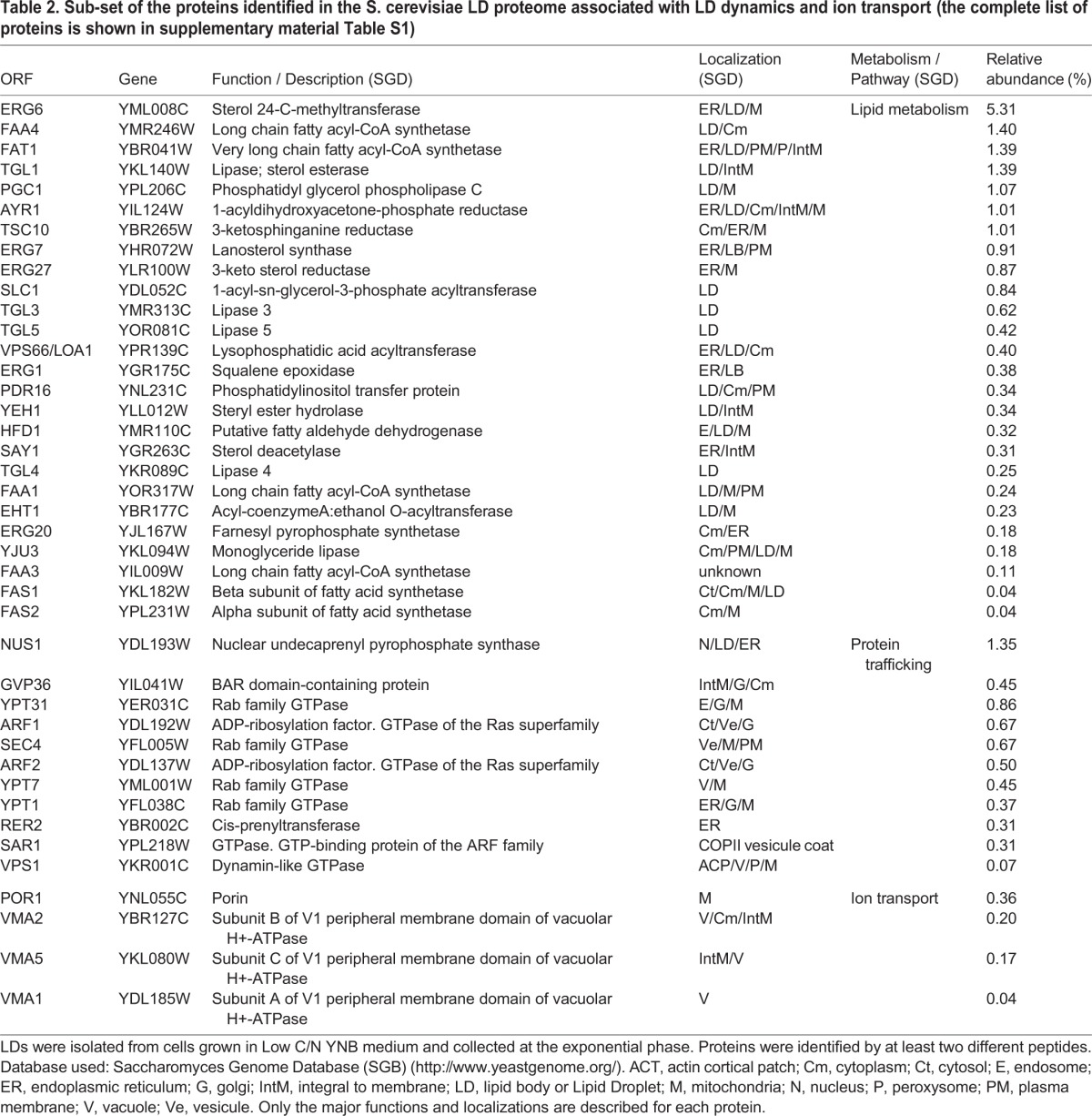
**Sub-set of the proteins identified in the S. cerevisiae LD proteome associated with LD dynamics and ion transport (the complete list of proteins is shown in supplementary material Table S1)**

### Transmission electron microscopy

Cells (∼10 A_600 nm_ U or 5 mg dry weight) were treated as previously described (Froissard et al., 2009). Thin sections (70 nm) were cut, stained with lead citrate and examined in a Zeiss EM902 TEM at 80 kV. TEM observations were made with a Zeiss EM902 transmission electron microscope, micrographs were acquired using a MegaView III CCD camera and analyzed with ITEM software (Eloise SARL, France).

### Fluorescence microscopy

GFP and RFP fluorescence were monitored using the microscope in cultured cells without further treatment. For Bodipy staining, cells were incubated for 10 min at room temperature with Bodipy 493/503 at 1.25 µg.ml^−1^ final concentration. For Hoechst staining, cells were incubated for 30 min at 28°C with Hoechst at 5 µg.ml^−1^ final concentration with shaking. After centrifugation for 30 s, the pellet was washed with water, then centrifuged for 30 s and recovered in Phosphate Buffered Saline (PBS). Cells were observed with the appropriate filters for epifluorescence (EPI) or the appropriate laser for confocal microscopy. Epifluorescence observations were made with a Zeiss Axio Imager microscope with fluorescence and Nomarski optics, images were acquired using a Roper CoolSnap HQ2 camera coupled to a Zeiss AxioVision driver (Carl Zeiss SAS, Marly le Roy, France). Confocal fluorescence observations were made with a Leica SP2 AOBS 405 and images were acquired using a LCS station Transmission Electron (Leica Microsystemes SAS, Nanterre, France).

### Fluorescence analysis

The fluorescence (485/518) of yeast strains was measured using a Fluoroskan Ascent Microplaque Fluorometer (Thermo Fisher Scientific, Courtaboeuf, France). Yeasts were grown in YNB with low C/N until the early stationary phase. Cells were stained with Bodipy 493/503 as previously described, or not for auto-fluorescence measure. A range of dilutions from 0 to 20 OD units was made and cells were transferred into a 96 white microwell plate. Auto-fluorescence was subtracted from the signal.

### Concanamycin A and BCECF-AM experiments

Yeasts were grown in YNB with low C/N until the exponential phase. For FA analysis by GC, OD was adjusted to 1 and cells were incubated under agitation for 30 min at 28°C with 1 µM concanamycin (in DMSO) or 0.1% (v/v) DMSO. They were then washed with water and freeze dried for 72 hours. To control the action of concanamycin A, a part of cells were adjusted to 1 OD and incubated under agitation for 30 min at 28°C with 18 µM BCECF-AM (in DMSO) or 0.2% DMSO before concanamycin A treatment. They were then observed with the appropriate filter by EPI.

### SDS-PAGE, gel staining and western blotting

For SDS-PAGE, proteins were run on precast 10% or 12% Bis-Tris polyacrylamide gels, using 3-(N-morpholino)propane sulfonic acid running buffer with anti-oxidant according to the manufacturer's instructions (Thermo Fisher Scientific). Molecular weight markers (Seeblue® prestained standard for Coomassie staining or MagicMark™ XP Western protein Standard for Chemiluminescence) were supplied by Thermo Fisher Scientific. Proteins were stained by incubating the gel overnight with 2 volumes of 0.4% (w/v) Coomassie Brilliant Blue G-250 in ethanol and 8 volumes of 2% (w/v) orthophosphoric acid and 10% (w/v) ammonium sulfate (Neuhoff et al., 1988). For analysis of Ypt7p partners from Co-IP, gels were also stained with silver nitrate according to the manufacturer's instructions (GE-Healthcare, Piscataway, USA), except that glutaraldehyde was omitted, and formaldehyde was only used in the development step. For immunoblot analysis, gels were blotted onto polyvinylidene difluoride (PVDF) membranes (Millipore, St-Quentin-en-Yvelines, France) under semidry conditions using a protocol described previously (Laurière, 1993).

### LD purification and total protein extraction

LDs from the GFP-YPT7/ERG6-RFP strain were separated by density gradients as previously described (Yu et al., 2000), and as optimized previosuly (Vindigni et al., 2013). Cells (400 ml of culture at 2 A_600 nm_ U.ml^−1^ in YNB Low C/N) were harvested by centrifugation at 3000 ***g*** for 10 min at 4°C. The pellet was washed and resuspended in 3 ml of Fat Body Buffer (10 mM HEPES, 10 mM KCl, 0.1 mM EDTA, 0.1 mM EGTA, pH 7.5) (FBB) supplemented with anti-protease. Cells were disrupted in a One Shot Cell Disrupter (Constant System LDT, Daventry, UK) at a maximum pressure of 2.5 kbars. After centrifugation, the volume of cleared extract was mixed with an equal volume of FBB including 1.08 M sucrose, transferred to an 11 ml ultracentrifugation tube and overlaid with three successive layers of 0.27, 0.135 and 0 M sucrose buffered with FBB. After ultracentrifugation at 150,000 ***g*** for 90 min at 4°C, 400 µl of floating LDs were recovered from the top of the gradient. For SDS-PAGE analysis, proteins from purified LDs were precipitated overnight with 10% (w/v) trichloroacetic acid (TCA) in an ice bath and re-suspended in SDS-PAGE buffer.

A total protein extract was prepared using the NaOH/TCA lysis technique as previously described (Volland et al., 1994).

### Immunoblot experiments

PVDF membranes were first saturated for 1 h in PBS, 0.05% (w/v) Tween 20 (PBST) containing 3% (w/v) milk powder (PBST-L). Membranes were then incubated for 1 h 30 with the primary antibody diluted in the same buffer. Mouse monoclonal antibody to GFP was from Roche Diagnostics (Meylan, France), mouse antibody to beta-Actin was from Abcam (Paris, France), Rabbit antibody to DsRed fluorescent protein (RFP) was from Clonetech (St-Germain-en-Laye, France), goat antibody to Vam3(yN-18) was from Santa Cruz Biotechnology, Inc (Santa Cruz, USA). Rabbit antibody to gas1 was a generous gift from Professor H. Riezman (Basel University, Biozentrum, CH - 4056 Basel, Switzerland). After three washing steps, the membranes were incubated for 1 h 30 with the conjugate diluted in PBST-L. Goat antibodies to mouse-IgG or rabbit-IgG conjugated to horseradish peroxidase (HRP) were from Sigma-Aldrich Chimie (St. Louis, USA), donkey antibody to goat-IgG conjugated to HRP was from SouthernBiotech (Birmingham, USA). After five washing steps, membranes were incubated for 5 min with the SuperSignal West Dura Extended duration substrate (Thermo Scientific, Pierce Biotechnology, Rockford, USA). All washing steps lasted 10 min each in PBST. All steps were performed under gentle rocking at room temperature. Antibodies were diluted according to the manufacturer's instructions. Chemiluminescence was recorded using the luminescent image analyzer LAS-3000 supplied by Fujifilm (Tokyo, Japan).

### Co-IP experiments

Proteins from purified LDs (15 µg) solubilized in FBB buffer were extracted for 2 h on a rotary agitator in lysis buffer [anti-protease, 20 mM HEPES, 150 mM NaCl, 10% (v/v) glycerol and 1% (v/v) NP 40 final concentration]. After centrifugation at 20,000 ***g*** for 10 min, the supernatant was added to washed GFP-trap coupled to magnetic particles (GFPm) from ChromoTek GmbH (Planegg-Martinsried, Germany) and incubated for 2 h on a rotary agitator. The GFPm were gently washed twice with lysis buffer. Proteins were eluted successively by vortexing for 2 min at room temperature with 50 mM glycine pH 2.8, 0.65% (v/v) Tween 20 then by incubating for 10 min at 90°C with 1× LDS sample buffer. All steps, except where mentioned, were performed at 4°C. Glycine-eluted proteins were mixed with 0.5 volumes of Basic Buffer (BB) and boiled for 5 min before SDS-PAGE. LDS-eluted proteins were directly analyzed by SDS-PAGE. LDs were purified from either the GFP-YPT7/ERG6-RFP strain for the assay, or from the WT-p416GFP strain as a control of non-specific adsorption of proteins on GFPm. A soluble extract of GFP from the WT-p416GFP strain was also processed in the same way as the assay as a control for binding with the GFP part of GFP-Ypt7p to avoid identifying GFP rather than Ypt7p partners.

### Mass-spectrometry of proteins

For LD proteomic analysis, proteins from purified LDs (15 µg) were TCA-precipitated as described above and 15 µl of BB were added to the pellet. Proteins were heated at 37°C for 10 min in a water bath and loaded on a SDS gel. Electrophoresis was stopped when proteins had migrated 1 cm. After Coomassie staining, the entire strip was cut in small pieces, rinsed with ethanol and left to dry at room temperature. Dried samples were reduced, alkylated, trypsin-hydrolyzed and analyzed using a nanoLC-LTQ-Orbitrap. Proteins were validated when at least two distinct peptides were identified. For mass-spectrometry analysis of YPT7p partners from Co-IP, eluted proteins were run on an SDS gel and stained with Coomassie then silver nitrate. Bands of interest were cut out and destained with 15 mM potassium hexacyanoferrate III, 50 mM sodium thiosulfate for 30 min then washed with 25 mM ammonium bicarbonate, 50% (v/v) acetonitrile for 15 min. The solution was discarded and acetonitrile was added to the bands which were dried under vacuum. Dried samples were analyzed by LC-MS/MS as previously described.

### Fatty acid analysis

Cells (100 ml of culture at 5 A_600 nm_ U.ml^−1^) were collected by centrifugation, washed with water and freeze dried for 72 h. Samples (20 mg dried cells) were processed as previously described (Froissard et al., 2009) and analyzed by gas chromatography (GC) using a 7890A chromatograph with a Factor Four VF-23ms 30 m×0.25 mm capillary column (Agilent Technology, Santa-Clara, USA). The quantification was made by flame ionization detection at 270°C. The amount of each FA was calculated from the ratio between the FAME peak area and the heptadecanoic acid methyl ester peak area and expressed relative to cell dry weight.

## Supplementary Material

Supplementary Material
