# Supplementary material for: Regulation of lipid droplet dynamics in Saccharomyces cerevisiae depends on the Rab7-like Ypt7p, HOPS complex and V1-ATPase
Source: Biol Open. 2015 May 6;4(7):764–75. doi: 10.1242/bio.20148615 (PMC4571102; doi:10.1242/bio.20148615)
Supplement: Supplementary Material [file supp_4_7_764__index.html]

Regulation of lipid droplet dynamics in Saccharomyces cerevisiae depends on the Rab7-like Ypt7p, HOPS complex and V1-ATPase — Regulation of lipid droplet dynamics in Saccharomyces cerevisiae depends on the Rab7-like Ypt7p, HOPS complex and V1-ATPase — Supplementary Material 

# Regulation of lipid droplet dynamics in *Saccharomyces cerevisiae* depends on the Rab7-like Ypt7p, HOPS complex and V1-ATPase

## BIO20148615 Supplementary Material

- Supplementary Material
