# Supplementary material for: Regulation of lipid droplet dynamics in Saccharomyces cerevisiae depends on the Rab7-like Ypt7p, HOPS complex and V1-ATPase
Source: Biol Open. 2015 May 6;4(7):764–75. doi: 10.1242/bio.20148615 (PMC4571102; doi:10.1242/bio.20148615)
Supplement: Supplementary Material [file supp_bio.20148615_BIO20148615supp.pdf]

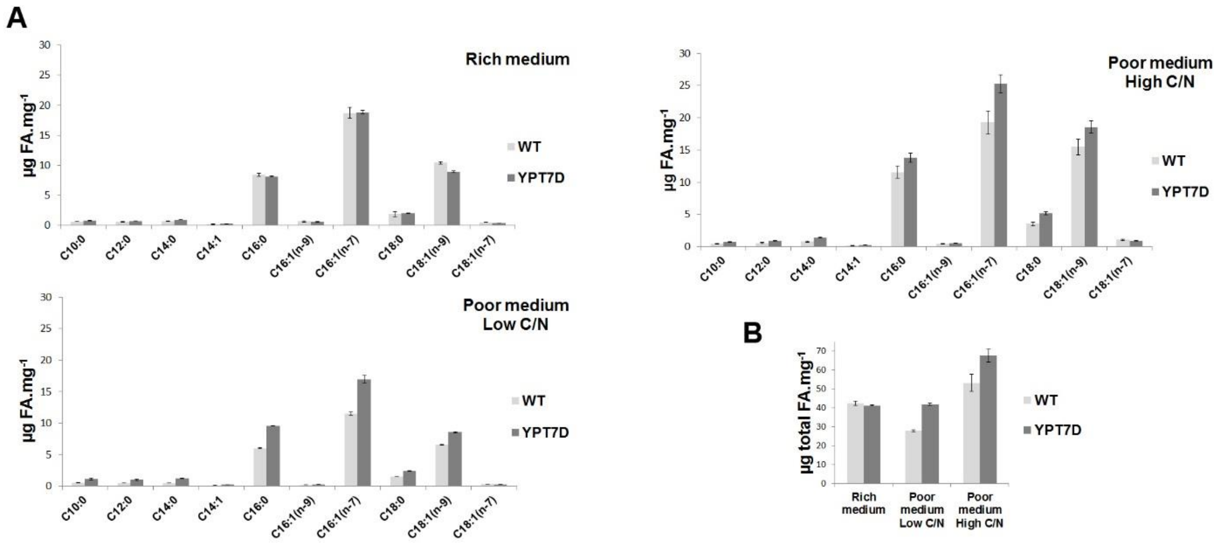

Fig S1. FA accumulation induced by Ypt7p defect depends on nutrition conditions. (A) Cells from *ypt7*Δ and WT strains were grown in rich medium (YPG) or poor medium (YNB low C/N or YNB high C/N) and processed for fatty acid methyl esters (FAME) production. Samples of the organic phase were separated by GC, and FAME identification was based on their retention time. The amount of each class of FA was expressed in µg of FA per mg of dry weight. (B) The total amount of FA accumulated in the three strains is shown for each type of medium.

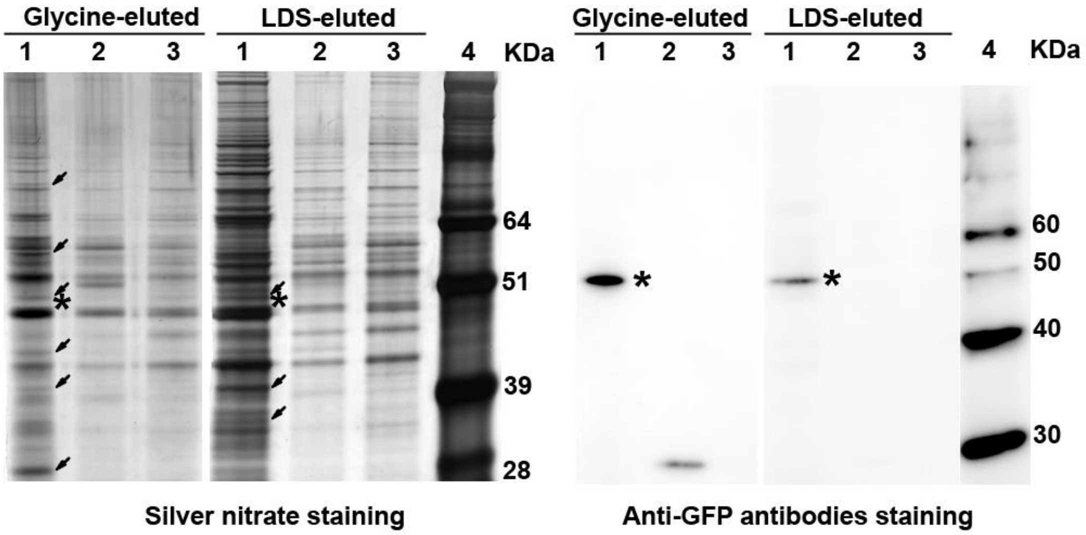

Fig S2. Co-immunoprecipitation experiments following by SDS-PAGE analysis of Glycine- and LDS-eluted proteins, either silver nitrate stained, or immunoblotted with anti-GFP antibodies. Lane 1: Assay (purified LDs from GFP-YPT7/ERG6-RFP; Lane 2: Control of GFP partners (soluble extract of proteins WT-p416GFP); Lane 3: Control of non-specific adsorption of proteins on GFPm (purified LDs from WT-p416GFP); Lane 4: Molecular weight marker. Arrows indicate the bands which were cut out of the gel and processed for mass-spectrometry analysis as described in the Materials and Methods. Stars indicate the position of GFP-Ypt7p.

Table S1. Complete proteome of *S. cerevisiae* LDs

ORF	Gene	Function / Description (SGD)	Localization (SGD)	Metabolism / Pathway (SGD)	Relative abundance (%)
ACT1	YFL039C	Actin	Ct	Cytoskeleton	0,29
ADE1	YAR015W	SAICAR synthetase	Cm/N	Nucleotide metabolism	0,45
ADE16	YLR028C	AICAR transformylase and IMP cyclohydrolase	Ct	Nucleotide metabolism	0,08
ADE17	YMR120C	Bifunctional purine biosynthesis protein ADE17	Ct/PM	Nucleotide metabolism	0,13
ADE5,7	YGL234W	Bifunctional purine biosynthetic protein ADE5,7	Cm	Nucleotide metabolism	0,29
ADE6	YGR061C	Formylglycinamidine-ribonucleotide synthetase	Cm	Nucleotide metabolism	0,07
ADH1	YOL086C	Alcohol dehydrogenase	Ct, PM	Amino acid metabolism; Glucose fermentation	1,82
ADH5	YBR145W	Alcohol dehydrogenase isoenzyme V	Cm/N	Amino acid metabolism; glucose fermentation	0,19
ADK1	YDR226W	Adenylate kinase	Cm/M	Nucleotide metabolism	0,18
ADO1	YJR105W	Adenosine kinase	Cm/N	Nucleotide metabolism	0,19
ALD6	YPL061W	Aldehyde dehydrogenase	Ct/M	Carbohydrate metabolism	0,39
ARF1	YDL192W	ADP-ribosylation factor; GTPase of the Ras superfamily	Ct/Ve/G	Protein trafficking	0,67
ARF2	YDL137W	ADP-ribosylation factor; GTPase of the Ras superfamily	Ct/Ve/G	Protein trafficking	0,50
ASC1	YMR116C	Guanine nucleotide-binding protein subunit beta-like protein	R/Cm	Signal tranduction	0,45
ATP1	YBL099W	ATP synthase	IntM/M	Ion transport	0,05
AYR1	YIL124W	1-Acyldihydroxyacetone-phosphate reductase	ER/LB/Cm/IntM/M	Lipid metabolism	1,01
CDC19	YAL038W	Pyruvate kinase	Ct, PM	Carbohydrate metabolism	2,02
CPR1	YDR155C	Cytoplasmic peptidyl-prolyl cis-trans isomerase	H/N/M	Amino acid metabolism	1,16
EFT1	YOR133W	Elongation factor two	R	Translation	1,02
EHT1	YBR177C	Acyl-coenzymeA:ethanol O-acyltransferase	LB/M	Lipid metabolism	0,23
ENO1	YGR254W	Enolase I	V/M/Cm/PM	Carbohydrate metabolism	1,75
ENO2	YHR174W	Enolase II	V/PM/M	Carbohydrate metabolism	3,11
ERG1	YGR175C	Squalene epoxidase	ER/LB	Ergosterol biosynthesis	0,38
ERG20	YJL167W	Farnesyl pyrophosphate synthetase	Cm/ER	Ergosterol biosynthesis	0,18
ERG27	YLR100W	3-keto sterol reductase	ER/M	Ergosterol biosynthesis	0,87
ERG6	YML008C	Sterol 24-C-methyltransferase	ER/LB/M	Ergosterol biosynthesis	5,31
ERG7	YHR072W	Lanosterol synthase	ER/LB/PM	Ergosterol biosynthesis	0,91
FAA1	YOR317W	Long chain fatty acyl-CoA synthetase	LB/M/PM	Fatty acid oxidation pathway	0,24
FAA3	YIL009W	Long chain fatty acyl-CoA synthetase	unknown	Fatty acid oxidation pathway	0,11
FAA4	YMR246W	Long chain fatty acyl-CoA synthetase	LB/Cm	Fatty acid oxidation pathway	1,40
FAS1	YKL182W	Beta subunit of fatty acid synthetase	Ct/Cm/M/LB	Lipid metabolism	0,04
FAS2	YPL231W	Alpha subunit of fatty acid synthetase	Cm/M	Lipid metabolism	0,04

FAT1	YBR041W	Very long chain fatty acyl-CoA synthetase	ER/LB/PM/P/IntM	Fatty acid oxidation pathway	1,39
FBA1	YKL060C	Fructose 1,6-bisphosphate aldolase	M/Ct/Cm	Carbohydrate metabolism	3,25
FPR1	YNL135C	Peptidyl-prolyl cis-trans isomerase	Cm/M/N	Amino acid metabolism	0,34
GND1	YHR183W	6-phosphogluconate dehydrogenase	Cm/M	Carbohydrate metabolism	0,11
GPM1	YKL152C	Glycerate phosphomutase	Ct/M	Carbohydrate metabolism	1,03
GRS1	YBR121C	glycyl-tRNA synthase	Cm/M	Transcription	0,07
GUA1	YMR217W	GMP synthase	unknown	Nucleotide metabolism	0,19
GUK1	YDR454C	Guanylate kinase	Cm/N	Nucleotide metabolism	0,27
GUS1	YGL245W	Glutamyl-tRNA synthetase	M/Cm	Amino acid metabolism	0,03
GVP36	YIL041W	BAR domain-containing protein	IntM/G/Cm	Protein trafficking	0,45
HFD1	YMR110C	Putative fatty aldehyde dehydrogenase	E/LB/M	Lipid metabolism	0,32
HOM6	YJR139C	Homoserine dehydrogenase	Cm/N	Amino acid metabolism	0,23
HSC82	YMR186W	ATPase; Hsp90 family; chaperone	Cm/M/PM	Chaperone / Stress proteins / Oxydoreductase	0,65
HSP82	YPL240C	ATPase; Hsp90 family; chaperone	C	Chaperone / Stress proteins / Oxydoreductase	0,56
HXK2	YGL253W	Hexokinase isoenzyme 2	Ct/M/N	Carbohydrate metabolism	0,54
HYP2	YEL034W	Translation elongation factor eIF-5A	R/Cm/M	Translation	0,54
ILV5	YLR355C	Acetohydroxyacid reductoisomerase and mtDNA binding protein	M	Amino acid metabolism	0,82
KAR2	YJL034W	ATPase; chaperone	ER	Chaperone / Stress proteins / Oxydoreductase	0,10
NUS1	YDL193W	Nuclear undecaprenyl pyrophosphate synthase	N/LB/ER	Proposed to be involved in protein trafficking	1,35
OLA1	YBR025C	P-loop ATPase	Cm	Unclassified	0,20
OSW5	YMR148W	Unknown function	IntM	Unclassified	0,81
OYE2	YHR179W	NADPH oxidoreductase	Cm/M/N	Chaperone / Stress proteins / Oxydoreductase	0,17
PDC1	YLR044C	Pyruvate decarboxylase	Ct/N	Amino acid metabolism	2,02
PDP1	YBR221C	E1 beta subunit of the pyruvate dehydrogenase complex	M	Unclassified	0,11
PDR16	YNL231C	Phosphatidylinositol transfer protein	LB/C/PM	Lipid metabolism	0,34
PET10	YKR046C	Unknown function	LB	Unclassified	1,52
PFK1	YGR240C	Phosphofructokinase	Cm/M	Carbohydrate metabolism	0,20
PGC1	YPL206C	Phosphatidyl glycerol phospholipase C	LB/M	Lipid metabolism	1,07
PGI1	YBR196C	PhosphoglucoIsomerase	Ct/M/PM	Carbohydrate metabolism	1,23
PGK1	YCR012W	3-phosphoglycerate kinase	M/Cm/PM	Carbohydrate metabolism	1,45
POR1	YNL055C	Mitochondrial porin	M	Ion transport	0,36
PSA1	YDL055C	GDP-mannose pyrophosphorylase	Cm	Carbohydrate metabolism; protein glycosylation	0,14
QA23_3395	nf	nf	nf	Unclassified	0,74
RER2	YBR002C	Cis-prenyltransferase	ER	Protein trafficking	0,31

RHO1	YPR165W	GTP-binding protein of the rho subfamily of Ras-like proteins	G/P/M/PM	Signal tranduction	0,19
RHR2	YIL053W	DL-glycerol-3-phosphatase	Cm/N	Lipid metabolism	0,67
RPL12A	YEL054C	Ribosomal protein of the large subunit	R	Ribosome	1,16
RPL16B	YNL069C	Ribosomal protein of the large subunit	R	Ribosome	0,30
RPL1A	YPL220W	Ribosomal protein of the large subunit	R	Ribosome	0,50
RPL20A	YMR242C	Ribosomal protein of the large subunit	R	Ribosome	0,50
RPL27A	YHR010W	Ribosomal protein of the large subunit	R	Ribosome	0,81
RPL4A	YBR031W	Ribosomal protein of the large subunit	R	Ribosome	0,20
RPL7A	YGL076C	Ribosomal protein of the large subunit	R/Cm	Ribosome	0,37
RPL7B	YPL198W	Ribosomal protein of the large subunit	R/Cm/N	Ribosome	0,24
RPL8A	YHL033C	Ribosomal protein of the large subunit	R	Ribosome	0,18
RPL9A	YGL147C	Ribosomal protein of the large subunit	R	Ribosome	0,76
RPP0	YLR340W	Ribosomal protein P0	R/Cm	Translation	1,20
RPS0A	YGR214W	Ribosomal protein of the small subunit	R	Ribosome	1,01
RPS10B	YMR230W	Ribosomal protein of the small subunit	R	Ribosome	1,01
RPS12	YOR369C	Ribosomal protein of the small subunit	R	Ribosome	0,67
RPS13	YDR064W	Ribosomal protein of the small subunit	R	Ribosome	0,45
RPS14A	YCR031C	Ribosomal protein of the small subunit	R	Ribosome	0,97
RPS16A	YMR143W	Ribosomal protein of the small subunit	R	Ribosome	0,27
RPS18A	YDR450W	Ribosomal protein of the small subunit	R/M	Ribosome	0,45
RPS20	YHL015W	Ribosomal protein of the small subunit	R	Ribosome	0,45
RPS21A	YKR057W	Ribosomal protein of the small subunit	R	Ribosome	0,67
RPS24A	YER074W	Ribosomal protein of the small subunit	R/M	Ribosome	0,54
RPS27A	YKL156W	Ribosomal protein of the small subunit	Cm/R	Ribosome	0,89
RPS28B	YLR264W	Ribosomal protein of the small subunit	R	Ribosome	1,35
RPS3	YNL178W	Ribosomal protein of the small subunit	R	Ribosome	1,44
RPS4A	YJR145C	Ribosomal protein of the small subunit	Cm/R	Ribosome	0,81
RPS5	YJR123W	Ribosomal protein of the small subunit	R	Ribosome	1,05
RPS6A	YPL090C	Ribosomal protein of the small subunit	R	Ribosome	0,57
RPS7A	YOR096W	Ribosomal protein of the small subunit	R	Ribosome	0,38
RPS8A	YBL072C	Ribosomal protein of the small subunit	R	Ribosome	0,38
RPS9A	YPL081W	Ribosomal protein of the small subunit	R/Cm	Ribosome	0,40
RVB2	YPL235W	ATP-dependent DNA helicase	N	Transcription	0,11
SAC6	YDR129C	Fimbrin	Actin cortical patch	cytoskeleton	0,10

SAH1	YER043C	S-adenosyl-L-homocysteine hydrolase	Cm	Amino acid metabolism	0,17
SAM1	YLR180W	S-adenosylmethionine synthetase	C	Amino acid metabolism	0,17
SAM2	YDR502C	S-adenosylmethionine synthetase	Unknown	Nucleotide metabolism	0,18
SAR1	YPL218W	GTPase, GTP-binding protein of the ARF family	COPII vesicule coat	Protein trafficking	0,31
SAY1	YGR263C	Sterol deacetylase	ER/IntM	Lipid metabolism	0,31
SCP160	YJL080C	RNA-binding G protein effector	ER	Unclassified	0,04
SCRG_02099	nf	6-phosphofructokinase	nf	Carbohydrate metabolism	0,23
SCY_4030	nf	nf	nf	Unclassified	0,54
SEC4	YFL005W	Rab family GTPase	Ve/M/PM	Protein trafficking	0,67
SHM2	YLR058C	Serine hydroxymethyltransferase	Cm/PM	Amino acid metabolism	0,11
SLC1	YDL052C	1-acyl-sn-glycerol-3-phosphate acyltransferase	LB	Lipid metabolism	0,84
SSA1	YAL005C	ATPase; stress-seventy subfamily A; chaperone	V/PM/Cm/N	Chaperone / Stress proteins / Oxydoreductase	1,23
SSA2	YLL024C	ATP binding protein; stress-seventy subfamily A	V/M/Ct/Cm/PM	Chaperone / Stress proteins / Oxydoreductase	1,52
SSA4	YER103W	Stress-seventy subfamily A	Cm/N	Chaperone / Stress proteins / Oxydoreductase	0,47
SSB1	YDL229W	ATPase; stress-seventy subfamily B; chaperone	C/PM	Chaperone / Stress proteins / Oxydoreductase	1,11
SSB2	YNL209W	ATPase; stress-seventy subfamily B; chaperone	C/PM	Chaperone / Stress proteins / Oxydoreductase	1,11
SSC1	YJR045C	Hsp70 family ATPase; stress-seventy subfamily C	M	Chaperone / Stress proteins / Oxydoreductase	0,83
SSE1	YPL106C	ATPase; Hsp90; chaperone	Cm	Chaperone / Stress proteins / Oxydoreductase	0,36
SSE2	YBR169C	HSP70	Cm	Chaperone / Stress proteins / Oxydoreductase	0,07
SSZ1	YHR064C	Hsp70 protein	Cm	Chaperone / Stress proteins / Oxydoreductase	0,29
SUB2	YDL084W	Component of the TREX complex	N	Transcription	0,14
TAL1	YLR354C	Transaldolase	Cm/N	Carbohydrate metabolism	0,21
TDH1	YJL052W	Glyceraldehyde-3-phosphate dehydrogenase	Cm/LB/M/PM	Carbohydrate metabolism	0,71
TDH2	YJR009C	Glyceraldehyde-3-phosphate dehydrogenase	Cm/LB/M/PM	Carbohydrate metabolism	1,52
TDH3	YGR192C	Glyceraldehyde-3-phosphate dehydrogenase	Cm/LB/M/PM	Carbohydrate metabolism	1,73
TEF1	YPR080W	Translation elongation factor 1A	Cm/M/R	Translation	0,89
TEF4	YKL081W	Translational elongation factor eEF1B	R/M	Translation	0,56
TGL1	YKL140W	Lipase; sterol esterase	LB/IntM	Lipid metabolism	1,39
TGL3	YMR313C	Lipase 3	LB	Lipid metabolism	0,62
TGL4	YKR089C	Lipase 4	LB	Lipid metabolism	0,25
TGL5	YOR081C	Lipase 5	LB	Lipid metabolism	0,42
TIF1	YKR059W	Translation initiation factor eIF4A	R	Translation	0,73
TPI1	YDR050C	Triose phosphate isomerase	M/Cm/PM	Carbohydrate metabolism	1,35
TSC10	YBR265W	3-ketosphinganine reductase	Cm/ER/M	Lipid metabolism	1,01

TY1B	nf	nf	nf	Unclassified	0,04
VIN13_1550	nf	nf	nf	Unclassified	0,60
VMA1	YDL185W	Subunit A of the V1 peripheral membrane domain of vacuolar H+-ATPase	V	Ion transport	0,04
VMA2	YBR127C	Subunit B of the V1 peripheral membrane domain of vacuolar H+-ATPase	V/Cm/IntM	Ion transport	0,20
VMA5	YKL080W	Subunit C of the V1 peripheral membrane domain of vacuolar H+-ATPase	IntM/V	Ion transport	0,17
VPS1	YKR001C	Dynamin-like GTPase	actin cortical patch/V/P/M	Protein trafficking	0,07
VPS66/LOA1	YPR139C	Lysophosphatidic acid acyltransferase	ER/LB/Cm	Lipid metabolism	0,40
YDR341C	YDR341C	Arginyl-tRNA synthetase	Cm	Amino acid metabolism	0,07
YEF3	YLR249W	Translational elongation factor eEF1B	R	Translation	0,41
YEH1	YLL012W	Steryl ester hydrolase	LB/IntM	Lipid metabolism	0,34
YHB1	YGR234W	Nitric oxide oxidoreductase	Ct/M/Cm	Chaperone / Stress proteins / Oxydoreductase	0,18
YJU3	YKL094W	Monoglyceride lipase	Cm/PM/LB/M	Lipid metabolism	0,18
YMR147W	YMR147W	Unknown function	-	Unclassified	1,20
YOR059C	YOR059C	Unknown function	LB	Unclassified	0,39
YOR246C	YOR246C	late endosome and Vacuole interface function; oxydoreductase activity	V/LB	Chaperone / Stress proteins / Oxydoreductase	1,35
YPR147C	YPR147C	Unknown function	Cm	Unclassified	1,16
YPT1	YFL038C	Rab family GTPase	ER/G/M	Protein trafficking	0,37
YPT31/8	YER031C	Rab family GTPase	E/G/M	Protein trafficking	0,86
YPT7	YML001W	Rab family GTPase	V/M	Protein trafficking	0,45

LDs were isolated from cells grown in Low C/N YNB medium and collected at the exponential phase. Proteins were identified by at least two different peptides. Database used: Saccharomyces Genome Database (SGB) (http://www.yeastgenome.org/). ACT, actin cortical patch; Cm, cytoplasm; Ct, cytosol; E, endosome; ER, endoplasmic reticulum; G, golgi; IntM, integral to membrane; LD, lipid body or Lipid Droplet; M, mitochondria; N, nucleus; P, peroxysome; PM, plasma membrane; V, vacuole; Ve, vesicule. Only main functions and localizations were described for each protein.

Table S2. Complete list of potential Ypt7p partners identified by LC-MS/MS

Band (approximate apparent MW)	ORF	Gene	Function / Description (SGD)	Localization (SGD)	Metabolism / Pathway (SGD)	PAI
85 KDa Glycine elution	MET6	YER091C	Methyltetrahydropteroyltriglutamate-homocysteine methyltransferase	Cm/PM	Amino acid metabolism	0,4
63 KDa Glycine elution	TGL1	YKL140W	Lipase; Sterol esterase	LB/IntM	Lipid metabolism	0,1
51 kDa Glycine elution	YSC84	YHR016C	Actin-binding protein	Actin cortical patch	Cytoskeleton	0,4
	GND1	YHR183W	6-phosphogluconate dehydrogenase	Cm/M	Carbohydrate metabolism	0,3
	YPT7	YML001W	Rab family GTPase	V/M	Trafficking protein	0,4
40 kDa Glycine elution	PEP4	YPL154C	Vacuolar aspartyl protease (proteinase A)	V/M	-	0,4
	LSP1	YPL004C	Member of the BAR domain family	Eisosome/Cm/M/PM	-	0,1
37 kDa Glycine elution	ADH1	YOL086C	Alcohol dehydrogenase	Ct, PM	Amino acid metabolism; Glucose fermentation	0,9
	TAL1	YLR354C	Transaldolase	Cm/N	Carbohydrate metabolism	0,2
30 kDa Glycine elution	PET10	YKR046C	Unknown function	LB	-	0,4
	POR1	YNL055C	Porin	M	Ion transport	0,3
51 kDa SDS elution	GND1	YHR183W	6-phosphogluconate dehydrogenase	Cm/M	Carbohydrate metabolism	0,9
	EHT1	YBR177C	Acyl-coenzymeA:ethanol O-acyltransferase	LB/M	Lipid metabolism	0,5
	YSC84	YHR016C	Actin-binding protein	Actin cortical patch	Cytoskeleton	0,6
	VMA13	YPR036W	Subunit H of the V1 peripheral membrane domain of vacuolar H+-ATPase	IntM/V	Ion transport	0,4
	YPT7	YML001W	Rab family GTPase	V/M	Trafficking protein	0,6
	KES1	YPL145C	Member of the oxysterol binding protein family	G/IntM/Cm	Trafficking protein	0,3
	YMR027W	YMR027W	Unknown function	Cm/N	-	0,2
	GDI1	YER136W	GDP dissociation inhibitor	Vesicule	Trafficking protein	0,3
	LYS20	YDL182W	Homocitrate synthase isozyme	N/M	Amino acid metabolism	0,2
	SER33	YIL074C	3-phosphoglycerate dehydrogenase	Cm	Amino acid metabolism	0,1
	FUM1	YPL262W	Fumarase	Ct/M	TAC cycle	0,2
	AIM17	YHL021C	Unknown function	M	-	0,1
37 kDa SDS elution	ADH1	YOL086C	Alcohol dehydrogenase	Ct, PM	Amino acid metabolism; Glucose fermentation	2,6
	PGK1	YCR012W	3-phosphoglycerate kinase	M/Cm/PM	Carbohydrate metabolism	0,8
	TAL1	YLR354C	Transaldolase	Cm/N	Carbohydrate metabolism	0,9
	HOM6	YJR139C	Homoserine dehydrogenase	Cm/N	Amino acid metabolism	0,8
	YPR127W	YPR127W	Unknown function	Ct/N	-	0,6
	TDH3	YGR192C	Glyceraldehyde-3-phosphate dehydrogenase	Cm/LB/M/PM	Carbohydrate metabolism	0,7
	ILV5	YLR355C	Acetohydroxyacid reductoisomerase and mtDNA binding protein	M	Amino acid biosynthesis	0,5
	GRE2	YOL151W	3-methylbutanal reductase	Cm/N	Lipid metabolism; response to stress	0,2
	ADO1	YJR105W	Adenosine kinase	Cm/N	Nucleotide metabolism	0,3
	MDH3	YDL078C	Malate dehydrogenase	P	Fatty-acid oxidation pathway	0,2

	IDH2	YOR136W	Isocitrate dehydrogenase	M	TAC cycle	0,2
	CDC10	YCR002C	Septin; GTPase	N	Cell Division Cycle	0,1
	FBA1	YKL060C	Fructose 1,6-bisphosphate aldolase	M/Ct/Cm	Carbohydrate metabolism	0,2
34 kDa SDS elution	RPP0	YLR340W	Ribosomal protein P0	R/Cm	Translation	1
	ASC1	YMR116C	Guanine nucleotide-binding protein subunit beta-like protein	R/Cm	Signal transduction	0,5
	ADH1	YOL086C	Alcohol dehydrogenase	Ct, PM	Amino acid metabolism; Glucose fermentation	0,4
	PET9	YBL030C	ADP/ATP carrier	IntM/M	-	0,4
	BMH2	YDR099W	14-3-3 protein	N/PM	Signal transduction	0,4
	IPP1	YBR011C	Inorganic pyrophosphatase	Ct	-	0,5
	AYR1	YIL124W	1-acyldihydroxyacetone-phosphate reductase	ER/LB/Cm/IntM/M	Lipid metabolism	0,3
	SNF4	YGL115W	Activating gamma subunit of the AMP-activated Snf1p kinase complex	Cm/N/PM	-	0,2
	TSC10	YBR265W	3-ketosphinganine reductase	Cm/ER/M	Lipid metabolism	0,3
	ERG27	YLR100W	3-keto sterol reductase	ER/M	Ergosterol biosynthesis	0,2
	PRE10	YOR362C	Proteinase yscE	Proteasome/ER/N/M	Protein metabolism	0,2
	PHB1	YGR132C	Subunit of the prohibitin complex; Chaperone	M	-	0,2
	CAP2	YIL034C	Beta subunit of the capping protein (CP) heterodimer	Actin cortical patch	Cytoskeleton	0,3

Proteins were identified by at least two different peptides. Database used: Saccharomyces Genome Database (SGB) (http://www.yeastgenome.org/). ACT, actin cortical patch; Cm, cytoplasm; Ct, cytosol; E, endosome; ER, endoplasmic reticulum; G, golgi; IntM, integral to membrane; LD, lipid body or Lipid droplet; M, mitochondria; N, nucleus; P, peroxysome; PM, plasma membrane; V, vacuole; Ve, vesicule. Only main functions and localizations were described for each protein.
